# Drug Abuse Research Trend Investigation with Text Mining

**DOI:** 10.1155/2020/1030815

**Published:** 2020-02-01

**Authors:** Li-Wei Chou, Kang-Ming Chang, Ira Puspitasari

**Affiliations:** ^1^Department of Physical Medicine and Rehabilitation, China Medical University Hospital, Taichung, Taiwan; ^2^Department of Physical Therapy, Graduate Institute of Rehabilitation Science, China Medical University, Taichung, Taiwan; ^3^Department of Rehabilitation, Asia University Hospital, Taichung, Taiwan; ^4^Department of Photonics and Communication Engineering, Asia University, Taichung 41354, Taiwan; ^5^Department of Medical Research, China Medical University Hospital, China Medical University, Taichung 40402, Taiwan; ^6^Information System Study Program, Faculty of Science and Technology, Universitas Airlangga, Surabaya, Indonesia

## Abstract

Drug abuse poses great physical and psychological harm to humans, thereby attracting scholarly attention. It often requires experience and time for a researcher, just entering this field, to find an appropriate method to study drug abuse issue. It is crucial for researchers to rapidly understand the existing research on a particular topic and be able to propose an effective new research method. Text mining analysis has been widely applied in recent years, and this study integrated the text mining method into a review of drug abuse research. Through searches for keywords related to the drug abuse, all related publications were identified and downloaded from PubMed. After removing the duplicate and incomplete literature, the retained data were imported for analysis through text mining. A total of 19,843 papers were analyzed, and the text mining technique was used to search for keyword and questionnaire types. The results showed the associations between these questionnaires, with the top five being the Addiction Severity Index (16.44%), the Quality of Life survey (5.01%), the Beck Depression Inventory (3.24%), the Addiction Research Center Inventory (2.81%), and the Profile of Mood States (1.10%). Specifically, the Addiction Severity Index was most commonly used in combination with Quality of Life scales. In conclusion, association analysis is useful to extract core knowledge. Researchers can learn and visualize the latest research trend.

## 1. Introduction

Because of the rapid development of information technology, information regarding various issues has been widely dispersed. Academia has long synthesized existing information and literature to acquire new knowledge by using a large amount of data. This is currently done by first integrating analytical results from individual studies through systematic reviews and meta-analyses and then conducting statistical analyses to develop general conclusions. The present method may elicit discussions about the causal relationships and descriptions in studies as well as proposals of alternating treatment design to extend the implications of the literature. For example, a systematic review of drug literature in 2008 intended to determine the prevalence of illicit drug injection among people aged 15–64 years and the prevalence of HIV among injecting drug users [1]. A previous study reviewed 11,022 questionnaires to estimate the prevalence of illicit drug use in 61 countries. The obtained results revealed that 77% of the population worldwide aged 15–64 years used illicit drugs, with China, the United States, and Russia having the most users. In addition, the study indicated that approximately 3.0 million people (range 0.8–6.6 million people) worldwide who use illicit drugs might be HIV positive. Another meta-analysis explored the relationship between drug use and the high prevalence of skin and soft tissue infection. Data of 20 papers involving 9,502 patients presented a high correlation between the two [2]. In addition to improving statistical methods, advancements in information technology have facilitated the development of artificial intelligence and big data algorithms, both of which have been extensively applied in various fields, particularly the fields of public health and biomedical information [3]. One of the numerous applications of big data is text mining. Based on natural language processing, this technique uses keyword matching and the connections between keywords to identify potentially useful information. Text mining has also been applied to biomedical research, rapidly extracting crucial information from a large amount of biomedical literature studies. Because automated screening makes reviews more efficient, numerous new tools have been introduced for text mining in biomedical research [4–6]. Nevertheless, this method is feasible only under the premise that researchers are proficient in determining the usability, applicability, adaptability, interoperability, and comparative accuracy of current text mining resources [7]. The existing research shows that text mining can reduce 30%–70% of the workload of literature review [8].

Text mining has various applications. For example, to facilitate the development of precision medicine, text mining has been applied to examination of electronic medical records. The extensive use of electronic medical records provides clinicians and researchers with large amounts of data, which can be transferred to effective clinical care tools [9]. Another example text mining application is the use of narrative text analysis of electronic medical records to explore adverse drug reactions (ADRs) [10]. Researchers also applied text mining to clinical progress notes of cardiovascular diseases; text mining enabled them to calculate the probability of developing said diseases. The said study reviewed 282,569 echocardiography reports to identify patients with trileaflet aortic stenosis (TAS) or coronary artery disease (CAD). The results revealed a positive predictive value of 0.95 compared with the standard of 0.53 by the International Classification of Diseases, Ninth Revision, Clinical Modification for TAS diagnosis and a positive predictive value of 0.97 compared with the standard of 0.86 for CAD diagnosis [11]. ADRs of using aesthetic medicine, ranging from severe morbidity to mortality, indicate the importance of drug safety. In the past, the lifecycle of a drug was monitored from drug development to clinical trials to detect safety problems at an early stage. The drug was continued to be monitored after marketing approval. The study also used text mining to identify potential safety concerns of drugs from source articles, including biomedical literature, articles posted by consumers on social media platforms, and narrative electronic medical records [12].

Applications of text mining can also be observed in drug and drug abuse research [13, 14]. For example, the study [15] developed a series of text mining procedures for designing new drugs. Using data from the DrugBank database, the said study aimed to determine how the chemical and protein compositions of drugs are related to disease-related genes and pathways to ultimately help develop new drugs [15]. In another study, text mining was employed to explore the relationship between drug abuse and depression among young adults using 17,723 abstracts downloaded from PubMed. During the text mining process, keywords from these abstracts were organized, and a keyword cloud was used to present the topic content directly and demonstrate the term distribution for each topic. The results demonstrated that the association between drug abuse and depression among young adults lies in the links between keywords—such as sexual experience and violence—as well as risk factors of substance use among young adults. Text mining is also commonly employed in neurological drug abuse research [16]. The National Institute of Statistics and Censuses of Argentina investigated the prevalence of psychoactive substances in the country to estimate their consumption of psychoactive substances [17]. A study in the UK employed text mining and big data techniques to investigate the effectiveness of varenicline as a pharmaceutical aid for smoking cessation. The aforementioned study employed association rule mining to analyze 46,685 individuals' data from the UK Health Improvement Network database. The results revealed that varenicline was most commonly prescribed to heavy smokers aged 31–60 years and those diagnosed with chronic obstructive pulmonary disease; varenicline was rarely prescribed to healthy people, people older than 60 years, light smokers, and smokers with mental illness or dementia [18]. Application of big data techniques to social networking data can also be used for drug abuse and addiction research [19]. A study examining the association between young adults and their nonmedical use of prescription medications analyzed 2,417,662 posts on Twitter. The said study discovered that 75.72% of tweets with URLs contained a hyperlink to an online affiliate marketer that links directly to illegal online pharmacies where Valium can be bought without a prescription [20].

The aforementioned literature demonstrates that big data techniques in various forms have already been applied to academic research regarding drug abuse. This study applied text mining to organize drug abuse literature with the objective of understanding the current trends of drug abuse research using big data and association analysis. The results may serve as references for researchers to quickly understand large amounts of existing knowledge within their field.

## 2. Materials and Methods

This study used the following keywords to search for and download drug abuse articles published till 2018 in PubMed: detoxification, addiction, drug abuse, substance, methadone, drug addiction, and therapy. EndNote, bibliographic management software, was employed to organize the collected literature. A total of 28,488 articles were collected. After filtering out duplicate articles, those without an abstract (title, keyword, year, and author), nonjournal articles, and those with general terms (e.g., background, objectives, methods, results, conclusions, stop words, and numbers), 19,843 articles remained. The bibliographic data were stored in Excel files. Article data included the journal name, article title, abstract, keywords, authors, and year of publication. Dissertation data were analyzed using PolyAnalyst (Megaputer Intelligence, Inc., Bloomington, IN, USA). The main computing functions of PolyAnalyst include data importing, data sorting, charting, classification, estimation, prediction, correlation, and clustering. The computing functions used in this study are text mining and link analyses [21]. The text mining tool has capability for scalability, visual creation of analysis, interactive visualization, drill-down analysis, and execution of reports. It also includes automatic spelling correction, search for words and terms, detection of unpredicted issue, and a dictionary editor for synonyms and abbreviations. It has several steps for data analysis, which are as follows:

1. Data loading: software process commands are written for text mining, functional nodes are connected to import the Excel files into PolyAnalyst, and the parameter types of the data are adjusted.
2. Spell check: the spelling correction is conducted to improve the accuracy of the data content, thereby reducing the deviation of the data mining result from the actual situation. This procedure belongs to data cleaning, data transformation, and text segmentation.
3. Keyword extraction: this step comprises two tabs. The first tab is for keyword extraction that comprises the investigated documents. It displays all records for a selected keyword with the word being highlighted. On the second tab, extraction is done to find phrases and stable combinations of words.
4. Link terms: after completing the preprocessing task, keyword extraction and link analysis are conducted. A huge amount of correlated keywords and phrases is connected with a graph by a given connection tension threshold. As we modified the threshold, low-tension relations are hidden and the graph updates to only display the remaining links. By increasing the minimum tension threshold, we filter out a small number of records where there is relation between two words.
5. Creating taxonomy: the term “taxonomy” is generally defined as a classification system. In the taxonomy, all custom categories are created by users underneath the root category.
6. Visualizing the categorization result: during analysis, we can see some results in the taxonomy following visualization.

**Figure 1:**
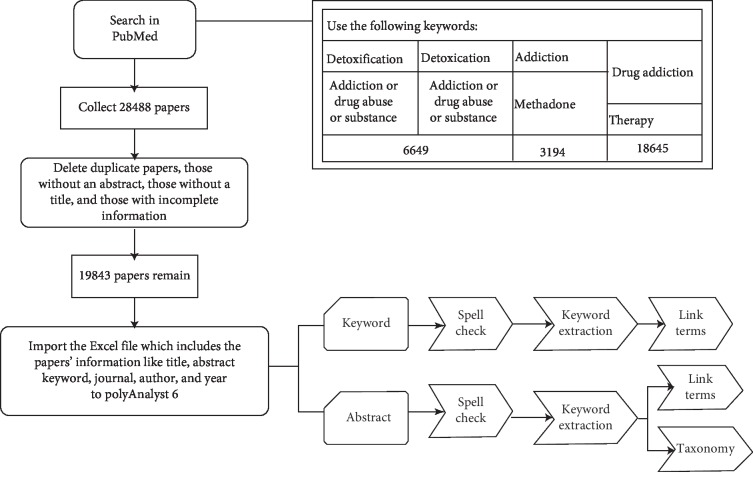
Experiment flowchart.

The full analysis process to determine the distributions of academic drug research is illustrated in Figure 1. The names of questionnaires commonly used in drug addiction treatment were extracted for text mining.

## 3. Results

**Figure 2:**
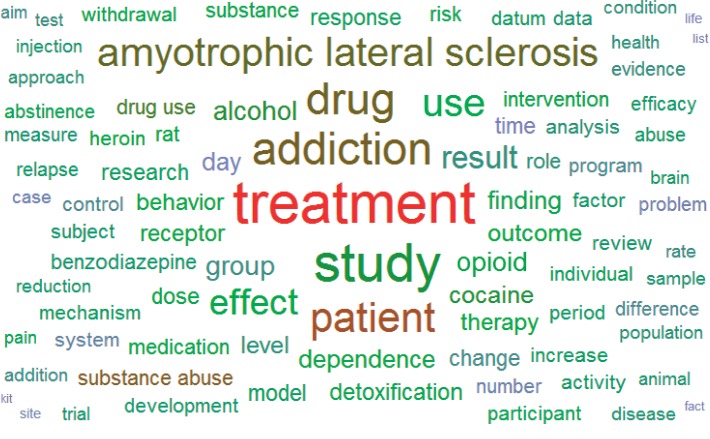
Keyword cloud.

**Figure 3:**
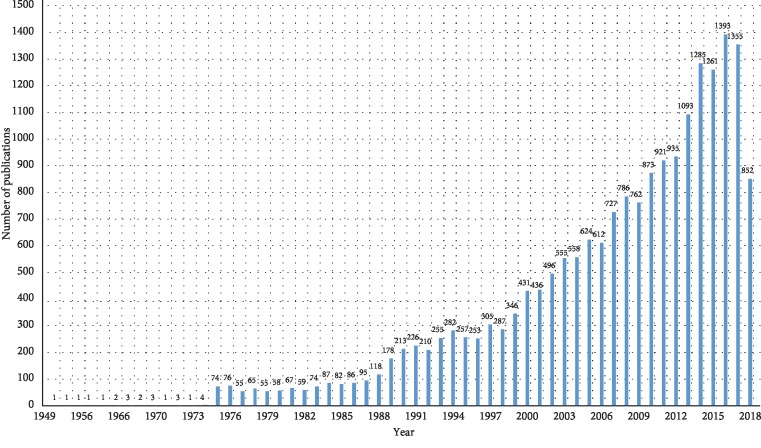
Publication distribution (by year).

**Figure 4:**
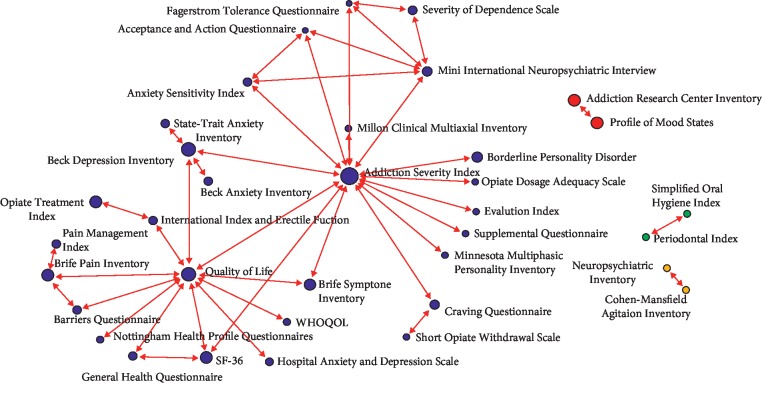
Associations pattern between questionnaires.

The distribution of collected keywords was visualized with a keyword cloud (Figure 2). More frequently a keyword appeared, the larger the area it occupied. In addition to the most frequently appearing keywords—treatment, study, addiction, drug, and patient—other terminologies related to drug addiction appeared. The numbers of dissertations in which these keywords appear are presented in Table 1. Arranging these publications by year resulted in the graph shown in Figure 3. Since 2013, the number of publications each year has exceeded 1,000, with the largest number of dissertations (1,393 papers) been published in 2016. Moreover, the number has increased with time, indicating that drug addiction treatment has received increasing attention from academia and suggesting the growth of future research. Analysis results revealed that, among all publications, 2,992 utilized questionnaires and scales. Sections discussing these measurement instruments were excerpted and organized. Questionnaires or scales used in more than 10 papers are presented in Table 2, which shows that the most commonly employed assessment tools were the Addiction Severity Index (ASI, 16.44%), Quality of Life (QoL, 5.01%) scales, and the Beck Depression Inventory (BDI, 3.24%).  Figure 4 shows a diagram of the link analysis of the questionnaires. Visualization of the association analysis reveals that two clusters formed, with the ASI and QoL scales as the respective cluster centroids. After further analysis of the association between questionnaires and the ASI as the cluster centroid, the most common questionnaires used in combination with the ASI were compiled into data shown in Table 3. Questionnaire combinations of the ASI with the QoL scale or the BDI were the most common assessment tools and the research direction most commonly approved by academia and clinical practitioners. For the second cluster with the QoL scale as the centroid, some of the questionnaires linked to it were also linked to the ASI cluster, whereas others were evidently linked to only the QoL cluster, such as studies utilizing the Brief Pain Inventory, General Health Questionnaire, and Brief Symptom Inventory.

## 4. Discussion

Although this article is implemented by packaged software POLY, it does not prevent others from using the ideas presented in this article. There are a variety of commercial software programs available to implement text mining, and one can also encode text mining by itself using a programming language such as R or Python. In addition, text mining for organizing references has numerous benefits, particularly speed. Visualization of data quickly gives researchers a comprehensive understanding of the development of academic research. Furthermore, link analysis reveals the associations between keywords and hidden information, both of which are unavailable through other standard research methods. This study chose to focus on the questionnaires used in drug addiction research. Researchers performing text mining may focus on other subject matter according to their needs. However, text mining does not guarantee notable results; results may also be ineffective. In addition, preprocessing to remove redundant text before text mining analysis is vital. Inadequate preprocessing may result in invalid keyword associations, leading to useless information. Conversely, excessive preprocessing can also remove useful information, which will not be presented in subsequent analyses. One research limitation of this study is the possibility of undiscovered questionnaires.

**Figure 5:**
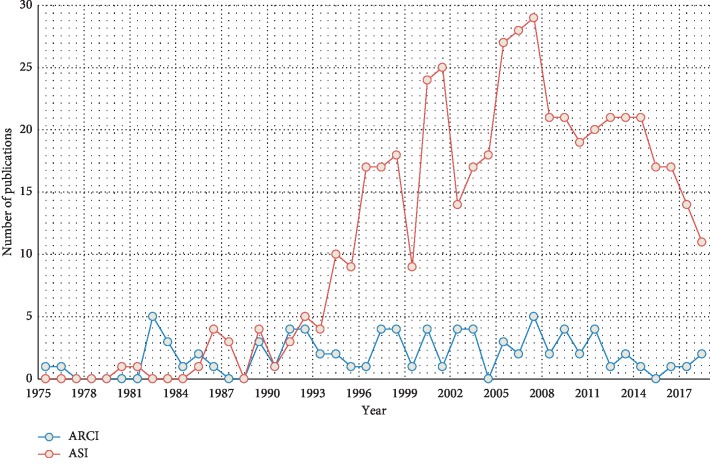
Publications with use of the ASI/ARCI by year.

The assessment questionnaires adopted by most studies were the ASI and Addiction Research Center Inventory (ARCI). Developed in 1980 [22], the ASI examines seven dimensions—the potential medical, employment/support status, alcohol, drug, legal, family/social, and psychiatric problem dimensions—and requires an interview lasting 50–70 minutes. The ARCI was developed in 1966 [23] and contains 550 true/false items. The numbers of publications with respective use of these two questionnaires are presented in Figure 5. Before 1995, the clinical use rates of the two were similar, but the use of the ASI has become more frequent than that of the ARCI since 1996. In addition to detecting the severity of drug addiction, the current research focuses on the physical and mental status, psychiatric assessments, sleep, and QoL of those addicted to drugs. Table 3 indicates that the six commonest questionnaires applied in combination with the ASI were QoL scales (*n* = 28), the BDI (*n* = 22), the Brief Symptom Inventory (*n* = 11), the Mini International Neuropsychiatric Interview (*n* = 6), the Craving Questionnaire (*n* = 4), and the SF-36 survey (*n* = 4). The first publication year, median publication year, and latest publication year of papers containing such questionnaire combinations are presented in Table 4. For example, the first publication year, median publication year, and most recent publication year of papers containing the combination of the ASI and the QoL scale (with the highest number compared with papers adopting other combinations) are 1998, 2009, and 2018, respectively. This shows that the aspects evaluated by QoL remain a topic of interest in academia. By contrast, the median publication year of papers containing the combination of the ASI and BDI is 2000, implying that the dimensions evaluated by the BDI are outdated, and therefore, it has received less attention in recent years. The questionnaire combination with the most recent median publication year (2012) was the ASI with the Mini International Neuropsychiatric Interview. However, this combination appeared in only six papers and was therefore deemed less pervasive than other combinations. The link analysis diagram in Figure 4 demonstrates that the Profile of Mood States was the assessment tool most frequently used in conjunction with the ARCI; this combination appeared in 19 papers. The first publication year, median publication year, and latest publication year of papers containing this combination are 1982, 1998, and 2012, respectively. This shows that the said combination was regarded as an effective assessment tool used in the early stage of relevant research and is currently rarely adopted by academia. Another drug addiction assessment questionnaire is the Severity of Dependence Scale (*n* = 18), which was frequently used in combination with the Mini International Neuropsychiatric Interview, but its use rate was 4% lower than that of the ASI.

## 5. Conclusion

This study used text mining to explore the use of questionnaires in drug addiction research. The visualization techniques used with text mining enable researchers to rapidly determine how frequently each questionnaire type appears in all relevant research and the numbers of employed assessment tools by year. Future studies may leverage this method to select promising assessment tools to explore topics of their interest.

## Figures and Tables

**Table 1:** Top 30 keywords and corresponding article numbers.

Ranking 1–10	No.	Ranking 11–20	No.	Ranking 21–30	No.
Treatment	11194	Alcohol	3793	Therapy	2794
Study	10138	Opioid	3647	Behavior	2751
Addiction	8134	Dependence	3236	Response	2598
Drug	7857	Day	3220	Time	2520
Patient	7278	Finding	3178	Change	2510
Amyotrophic lateral sclerosis	6833	Cocaine	2996	Dose	2475
Use	6727	Level	2969	Model	2475
Effect	6037	Outcome	2956	Research	2472
Result	4973	Receptor	2887	Rat	2425
Group	3844	Detoxification	2806	Role	2404

**Table 2:** Questionnaire distribution.

	Questionnaire	Publication no.	Ratio (%)
1	Addiction Severity Index	492	16.44
2	Quality of Life	150	5.01
3	Beck Depression Inventory	97	3.24
4	Addiction Research Center Inventory	84	2.81
5	Profile of Mood States	33	1.10
6	Craving Questionnaire	23	0.77
7	Brief Symptom Inventory	21	0.70
8	General Health Questionnaire	18	0.60
9	Severity of Dependence Scale	18	0.60
10	Brief Pain Inventory	18	0.60
11	Minnesota Multiphasic Personality Inventory	15	0.50
12	Short Opiate Withdrawal Scale	13	0.43
13	Opiate Treatment Index	13	0.43
14	SF-36	12	0.40
15	Young Mania Rating Scale	11	0.37
16	Hospital Anxiety and Depression Scale	11	0.37
17	Pittsburgh Sleep Quality Index	11	0.37
18	Neuropsychiatric Inventory	11	0.37
19	Temperament and Character Inventory	10	0.33
20	State-Trait Anxiety Inventory	10	0.33
21	Mini International Neuropsychiatric Interview	10	0.33
22	Childhood Trauma Questionnaire	10	0.33

**Table 3:** Commonest combinations of questionnaires.

	Questionnaire A	Questionnaire B	Tension	Support
1	Addiction Severity Index	Quality of Life	1.00	28
2	Addiction Severity Index	Beck Depression Inventory	0.99	22
3	Addiction Research Center Inventory	Profile of Mood States	0.88	19
4	Addiction Severity Index	Brief Symptom Inventory	0.67	11
5	Beck Depression Inventory	Quality of Life	0.26	11
6	Quality of Life	Brief Pain Inventory	0.39	7
7	Quality of Life	SF-36	0.33	7
8	Addiction Severity Index	Mini International Neuropsychiatric Interview	0.35	6
9	Quality of Life	General Health Questionnaire	0.35	5
10	Quality of Life	Brief Symptom Inventory	0.14	5
11	Addiction Severity Index	Craving Questionnaire	0.14	4
12	Addiction Severity Index	SF-36	0.11	4

**Table 4:** Combinations of the ASI or ARCI with other questionnaires and publication distributions.

		First published year	Median published year	Latest published year
*ASI*				
1	Quality of Life	1998	2009	2018
2	Beck Depression Inventory	1991	2000	2018
3	Brief Symptom Inventory	1995	2006	2018
4	Mini International Neuropsychiatric Interview	2005	2012	2015
5	Craving Questionnaire	2007	2009	2017
6	SF-36	2003	2004	2018

*ARCI*				
1	Profile of Mood States	1982	1998	2012

## Data Availability

Raw data are available at the following link: https://drive.google.com/file/d/1PiudHMsDhxUeT8-5EU_t9DTV_rXPFk5u/view?usp=sharing.
